# A novel prognostic signature based on smoking-associated genes for predicting prognosis and immune microenvironment in NSCLC smokers

**DOI:** 10.1186/s12935-024-03347-9

**Published:** 2024-05-15

**Authors:** Qixuan Li, Tianyi Wang, Yijie Tang, Xian Zou, Zhongqi Shen, Zixin Tang, Youlang Zhou, Jiahai Shi

**Affiliations:** 1 Nantong Key Laboratory of Translational Medicine in Cardiothoracic Diseases, and Research Institution of Translational Medicine in Cardiothoracic Diseases, Affiliated Hospital of Nantong University, Nantong, Jiangsu 226001 China; 2 Department of Thoracic Surgery, Affiliated Hospital of Nantong University, Nantong, Jiangsu 226001 China; 3 Medical School of Nantong University, Nantong, Jiangsu China; 4 Research Center of Clinical Medicine, Affiliated Hospital of Nantong University, Nantong, Jiangsu 226001 China; 5 School of Public Health, Nantong University, Nantong, Jiangsu 226001 China

**Keywords:** NSCLC, Prognostic signature, Immune microenvironment, Immune checkpoint, *FCGBP*

## Abstract

**Background:**

As a highly heterogeneous tumor, non-small cell lung cancer (NSCLC) is famous for its high incidence and mortality worldwide. Smoking can cause genetic changes, which leading to the occurrence and progress of NSCLC. Nevertheless, the function of smoking-related genes in NSCLC needs more research.

**Methods:**

We downloaded transcriptome data and clinicopathological parameters from Gene Expression Omnibus (GEO) databases, and screened smoking-related genes. Lasso regression were applied to establish the 7-gene signature. The associations between the 7-gene signature and immune microenvironment analysis, survival analysis, drug sensitivity analysis and enriched molecular pathways were studied. Ultimately, cell function experiments were conducted to research the function of *FCGBP* in NSCLC.

**Results:**

Through 7-gene signature, NSCLC samples were classified into high-risk group (HRG) and low-risk group (LRG). Significant difference in overall survival (OS) between HRG and LRG was found. Nomograms and ROC curves indicated that the 7-gene signature has a stable ability in predicting prognosis. Through the analysis of immune microenvironment, we found that LRG patients had better tumor immune activation. *FCGBP* showed the highest mutation frequency among the seven prognostic smoking related genes (*LRRC31*, *HPGD*, *FCGBP*, *SPINK5*, *CYP24A1*, *S100P* and *FGG*), and was notable down-regulated in NSCLC smokers compared with non-smoking NSCLC patients. The cell experiments confirmed that *FCGBP* knockdown promoting proliferation, migration, and invasion in NSCLC cells.

**Conclusion:**

This smoking-related prognostic signature represents a promising tool for assessing prognosis and tumor microenvironment in smokers with NSCLC. The role of *FCGBP* in NSCLC was found by cell experiments, which can be served as diagnostic biomarker and immunotherapy target for NSCLC.

**Supplementary Information:**

The online version contains supplementary material available at 10.1186/s12935-024-03347-9.

## Introduction

Ranking second in cancer incidence and first in cancer-related mortality, lung cancer causes about 700,000 deaths in China per year [1, 2]. Among lung cancer pathological subtypes, non-small cell lung cancer (NSCLC) was the most common pathological subtype, which accounts for almost 85% [3]. With the widespread application of new immunotherapy, surgical treatment and chemotherapy, NSCLC patients’ 5-year survival rate has gotten notable upgraded [4]. However, NSCLC patients are often diagnosed in the advanced stage, which is major cause of treatment failure and poor prognosis [5]. Therefore, exploring novel prognostic assessment and treatment options of NSCLC is indispensable.

Smoking is considered to be the most preventable cause of tumor occurrence and death [6]. The number of cancer deaths caused by smoking accounts for approximately one-third of all cancer deaths every year [7]. Numerous studies have proven that smoking will induce many cancers’ occurrence [8–13]. The change in smoking is paralleled by a change in the incidence of NSCLC [14]. Tobacco smoke contains more than 60 known or suspected carcinogens. Long-term smoking will inhibit the DNA repair mechanism and cause genetic changes, which lead to the occurrence and progression of cancer [15]. Due to gene changes precede obvious histopathological changes in tumor detection, it is urgent to identify the genetic changes of smoking and construct a new biomarker that can be used to stratify patients with NSCLC at the early stage. In recent years, it is a trend to construct new tumor biomarkers for NSCLC, and remarkable achievements have been achieved [16–19].

Herein, we identified differentially expressed smoking-related genes and constructed smoking-related prognostic signature to predict prognosis, TME, drug sensitivity and immunotherapeutic effect of NSCLC patients with smoking history. In addition, among differentially expressed smoking-related genes, we demonstrated the function of the highest mutation frequency gene *FCGBP* in NSCLC by cell experiments. Our results may yield a robust biomarker for assessing prognosis and tumor microenvironment of NSCLC patients with smoking history and immunotherapy response.

## Materials and methods

### Date preparation

In this study, NSCLC gene expression datasets were downloaded three cohorts (GSE50081, GSE68465 and GSE72094) from Gene Expression Omnibus (GEO; https://www.ncbi.nlm.nih.gov/geo/). Eliminating the data with incomplete smoking history and survival information, we obtained 106 NSCLC patients without smoking history and 736 NSCLC samples with smoking history. In addition, the copy number variation (CNV) frequency of somatic mutations was downloaded from the Cancer Genome Atlas (TCGA; https://portal.gdc.cancer.gov/) for genetic mutation analysis.

### Identification of differentially expressed smoking-related genes

To identifying smoking-related genes, we applied the ‘limma’ R package to identify DEGs between NSCLC smokers and NSCLC patients without smoking history. Genes met the conditions of log2 | FC |>1 and FDR < 0.05 are considered significant.

### Construction and validation of the prognostic signature

Applying univariate Cox regression analysis, prognostic smoking-related genes were filtered (*P* < 0.05). Then, using the R package ‘glmnet’, the least absolute contraction and selection operator (LASSO) Cox regression algorithm to minimize the risk of over fitting combining selected factors. Then, a 7-gene signature was constructed based on the screened prognostic smoking-related genes. Risk score was computed by the formula: risk score=∑ (gene × coefficient). NSCLC smokers were separated into high-risk group and low-risk group by the mid-value of the risk scores. To verify the 7-gene signature, external validation group by merging GSE29016 and GSE102287 was applied to test the performance of the signature in forecasting clinical results.

### Gene Set Enrichment Analysis (GSEA) and single sample Gene Set Enrichment analysis (ssGSEA)

Gene set “c2.cp.kegg.v7.4.symbols.gmt” from the MSigDB database (https://www.gsea-msigdb.org) was obtained to conduct GSEA. ssGSEA is an extension of the GSEA method, was applied to calculate the infiltration degree of 23 kinds of immune cells in each sample. Using the ‘GSEABase’ and ‘GSVA’R package, Differential expression related pathways and immune cells between LRG and HRG were identified. We simulated calculations 1000 times to obtain stable final data.

### Immune function analysis and immune checkpoint analysis

‘limma’, ‘GSVA’, ‘GSEABase’, ‘pheatmap’ and ‘reshape2’ R package were used to perform immune function analysis. The expression of immune checkpoints was determined through R package ‘limma’, ‘ggplot2’, ‘ggpubr’ and ‘reshape2’.

### Drug sensitivity analysis

The R package ‘gpubr’ and ‘pRRophetic’ R packages were used by us to explore chemotherapeutic drugs in different risk groups of NSCLC patients by calculating the half-inhibitory concentration (IC50) values of different drugs.

### NSCLC cell culture

Human NSCLC cell lines (A549 and NCI-H23) were purchased from the Shanghai Cell Bank, Chinese Academy of Sciences. NSCLC cell lines were cultured in the medium which consists of RPMI-1640 (Gibco, USA), 10% fetal bovine serum (Gibco, USA), 0.1 mg/ml streptomycin (Gibco, USA) and 100U/ml penicillin (Gibco, USA) and maintained under 37 °C at 5% CO_2_ atmosphere. Lentivirus targeting *FCGBP* knockdown was constructed by Hanbio Co. LTD (Shanghai, China). Target Seq: GGTGATCCATTCTGACTATGC.

### qPCR

The RNA-easy Isolation Reagent (R701, Vazyme, China) was used to extract total RNA from A549 and NCI-H23 cells. The HiScript III 1st Strand cDNA Synthesis Kit (R312, Vazyme, China) was used to synthetise cDNA. qPCR was applied to verity the knockdown efficiency of the synthesized shRNAs. qPCR was conducted by ChamQ Universal SYBR qPCR Master Mix (Q711, Vazyme, China) on the QuantStudio 5 Real-Time PCR Systems (A28569, Thermo Fisher Scientific, USA). Relative quantification was gotten by the 2^−ΔΔCT^ method. The primer used was as follows:

*FCGBP*-Forward primer: 5ʹ-GCAGTGAGTTCTCGTATGCTGAA-3ʹ;

*FCGBP*-Reverse primer: 5ʹ-GAAGGTGAGCAGTCCCAAGTT-3ʹ.

### Cell counting Kit-8 (CCK-8) assay

CCK8 assay was applied to detect A549 and NCI-H23 cells proliferation ability. 2000 transfected cells were inoculated in 96-well plates and added 10 µL of CCK-8 reagent (BS350A, Biosharp, China). The absorbance of cells at 450 nm was detected every 24 h for 5 days.

### Transwell assay

Precoat the Cell Culture Insert (353,097, BD Falcon, USA) with or without Matrix gel (356,234, Corning, USA) to evaluate the invasion or migration ability of cells respectively. 50,000 transfected cells were inoculated into the upper well. Adding serum free alkaline gel to the upper chamber, then we added the medium containing 20% FBS to the lower chamber in both assays. After 24 h of culture, the cells on the insert were fixed with polyformaldehyde, and finally dyed with crystal violet.

### Clone formation assay

500 transfected A549 and NCI-H23 cells were inoculated on the 6-well plate respectively. After incubating for 9 days, cells were fixed with paraformaldehyde and stained with crystal violet.

### Cell cycle and apoptosis analysis

Steps of cell cycle experiment: First, we fixed the cells in 70% alcohol overnight at 4 °C, washed them, and dyed them with propidium iodide (PI) containing RNase A (CA1510, Solarbio, China). Finally, the percentage of nsclc cell cycle phases (G0/G1, S and G2/M phases) was detected by flow cytometry (FACSCalibur, BD, USA). For apoptosis assay, cells washed with pre-cooled PBS and stained with Annexin V-Alexa Fluor 647/PI Apoptosis Assay Kit (FMSAV647, Fcmacs, China) by the manual. We used flow cytometry (FACSCalibur, BD, USA) to analyze the cells for apoptosis.

### Statistical analysis

The data is dealt with GraphPad Prism software and expressed as mean ± SD. The R 4.2.0 was applied to perform data processing.

## Results

### Identification of differentially expressed smoking-related genes in NSCLC

**Fig. 1:**
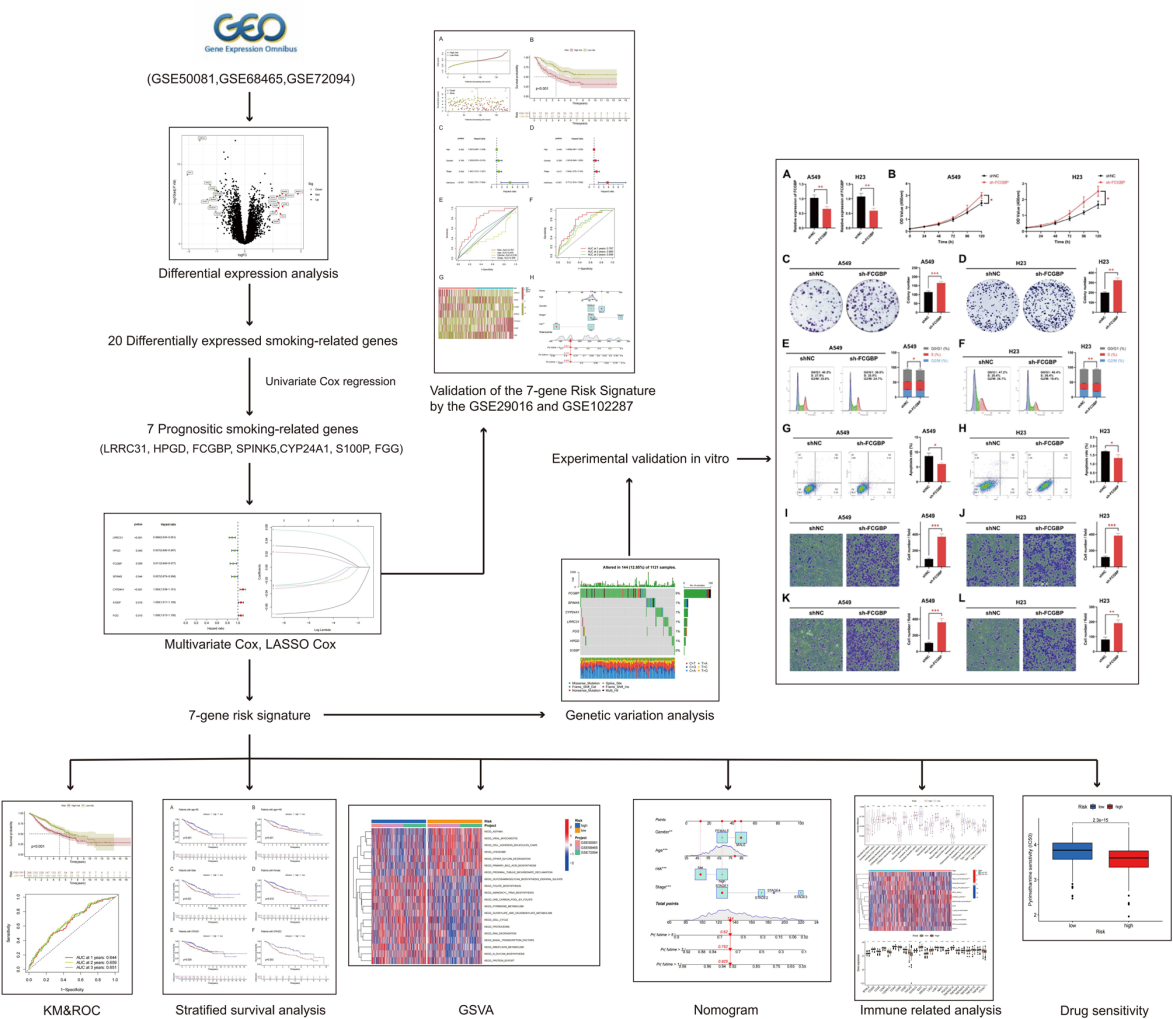
Research flow chart

**Fig. 2:**
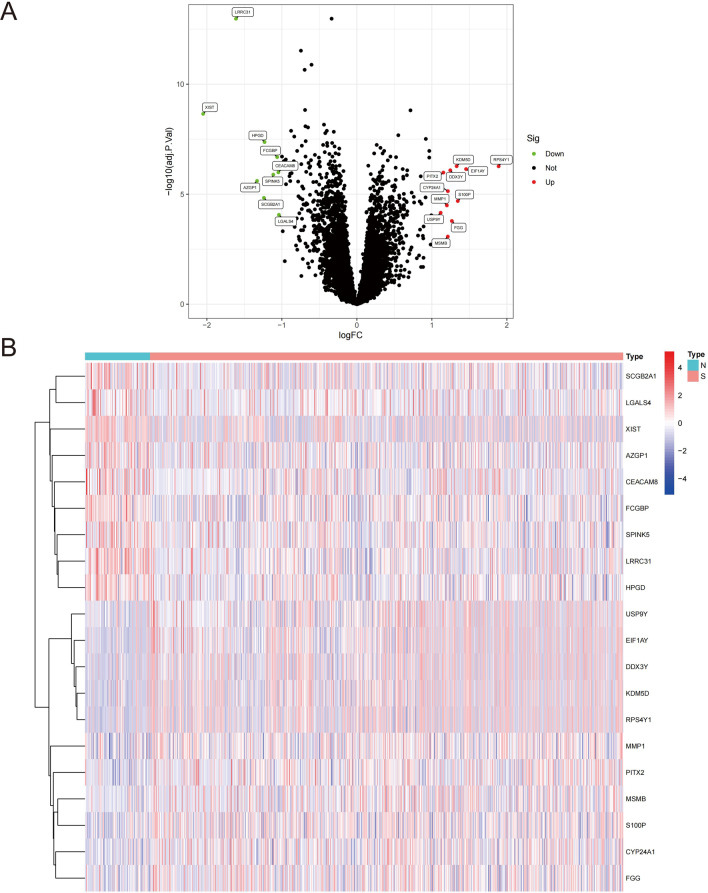
Identifying of differentially expressed smoking-related genes from GSE50081, GSE68465 and GSE72094. (**A**) Volcano plot of differentially expressed smoking-related genes by the conditions of log2 | FC |>1 and FDR < 0.05. (**B**) The heatmap of differentially expressed smoking-related genes expression landscape in smoking and non-smoking groups

The flowchart of the research is shown in Fig. 1. 842 NSCLC cases that came from GSE50081, GSE68465 and GSE72094. Deleting unqualified cases, we obtained 106 NSCLC patients without smoking history and 736 NSCLC patients with smoking history. The mRNA expression profiles of genes between NSCLC samples with smoking history and NSCLC samples without smoking history was analyzed. With the condition of *p* < 0.05 and |log2 FC| ≥ 1, there are 20 smoking-related genes were differentially expressed. Among these smoking-related genes, 11 genes were upregulated and 9 genes were downregulated (Fig. 2A). The expression landscape of the 20 differentially expressed smoking-related genes in smoking group and non-smoking group were illustrated in a heatmap (Fig. 2B).

### Construction of prognostic signature based on prognostic smoking-related genes for patient with smoking history

**Fig. 3:**
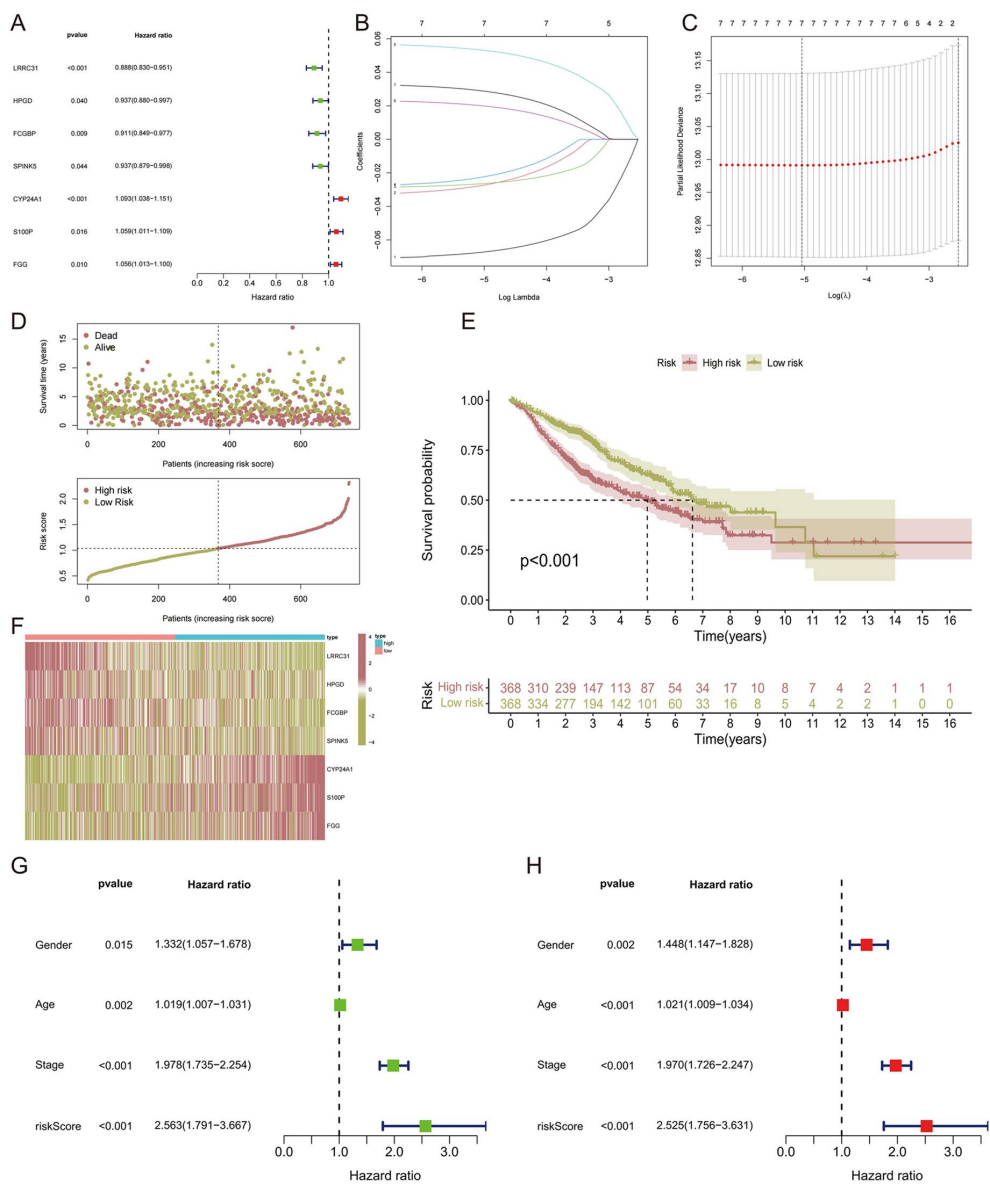
Establishing the 7-gene signature. (**A**) Forest plot of seven prognostic smoking related genes screened using Cox univariate regression analysis. (**B**, **C**) Cvift and lambda curves of LASSO regression applied with minimum criteria. (**D**) Risk scores and survival status of patients in two risk groups. (**E**) K-M curves of the OS of HRG and LRG. (**F**) Heatmap of the expression of seven prognostic smoking-related genes in HRG and LRG. (**G**, **H**) Univariate and multivariate Cox regression analysis of the 7-gene risk signature

Applying univariate Cox regression analysis, 7 genes (*LRRC31*, *HPGD*, *FCGBP*, *SPINK5*, *CYP24A1*, *S100P* and *FGG*) out of the 20 differentially expressed smoking-related genes were significantly correlated with overall survival (OS) of NSCLC patients with smoking history in GSE50081, GSE68465 and GSE72094 cohort (Fig. 3A). Lasso Cox regression was used to identify the key genes with the best prognostic value by reducing the dimension, and the relative coefficient of smoking related genes was calculated (Fig. 3B-C). The risk score is obtained by the following formula. Risk score = *LRRC31* exp. * (-0.071490275115065) + *HPGD* exp. * (-0.0338294194692034) + *FCGBP* exp. * (-0.0289843021911047) + *SPINK5* exp.* (-0.0289133244265656) + *CYP24A1* exp. * 0.0572743744393659 + *S100P* exp. * 0.0237236238563723 + *FGG* exp. * 0.0335046687925319. Then, by the median value of risk score, NSCLC patients with smoking history were separated in low-risk group (LRG) and high-risk group (HRG) (Fig. 3D). The clinical data of HRG and LRG patients are presented in Table 1. Then, conducting survival analysis, we found that HRG patients had a worse OS than those in LRG (Fig. 3E). A heatmap visualized the distribution of *LRRC31*, *HPGD*, *FCGBP*, *SPINK5*, *CYP24A1*, *S100P* and *FGG* in two groups (Fig. 3F). Next, univariate and multivariate Cox regression analyses demonstrated that the 7-gene risk signature is an independent prognostic indicator for predicting prognosis of NSCLC patients with smoking history (Fig. 3G-H).

**Table 1:** The clinical data of HRG and LRG patients

Covariates	Type	HRG	LRG
Age	<=65	152 (41.30%)	138 (37.5%)
Age	>65	214 (58.15%)	227 (61.68%)
Age	unknown	2 (0.55%)	3 (0.82%)
Gender	Female	176 (47.82%)	172 (46.74%)
Gender	Male	190 (51.63%)	193 (52.44%)
Gender	unknown	2 (0.55%)	3 (0.82%)
Stage	Stage I	225 (61.14%)	250 (67.93%)
Stage	Stage II	89 (24.18%)	71 (19.29%)
Stage	Stage III	48 (13.03%)	39 (10.60%)
Stage	Stage IV	4 (1.10%)	6 (1.63%)
Stage	unknown	2 (0.55%)	2 (0.55%)

### Clinical information evaluation of prognostic risk signature

**Fig. 4:**
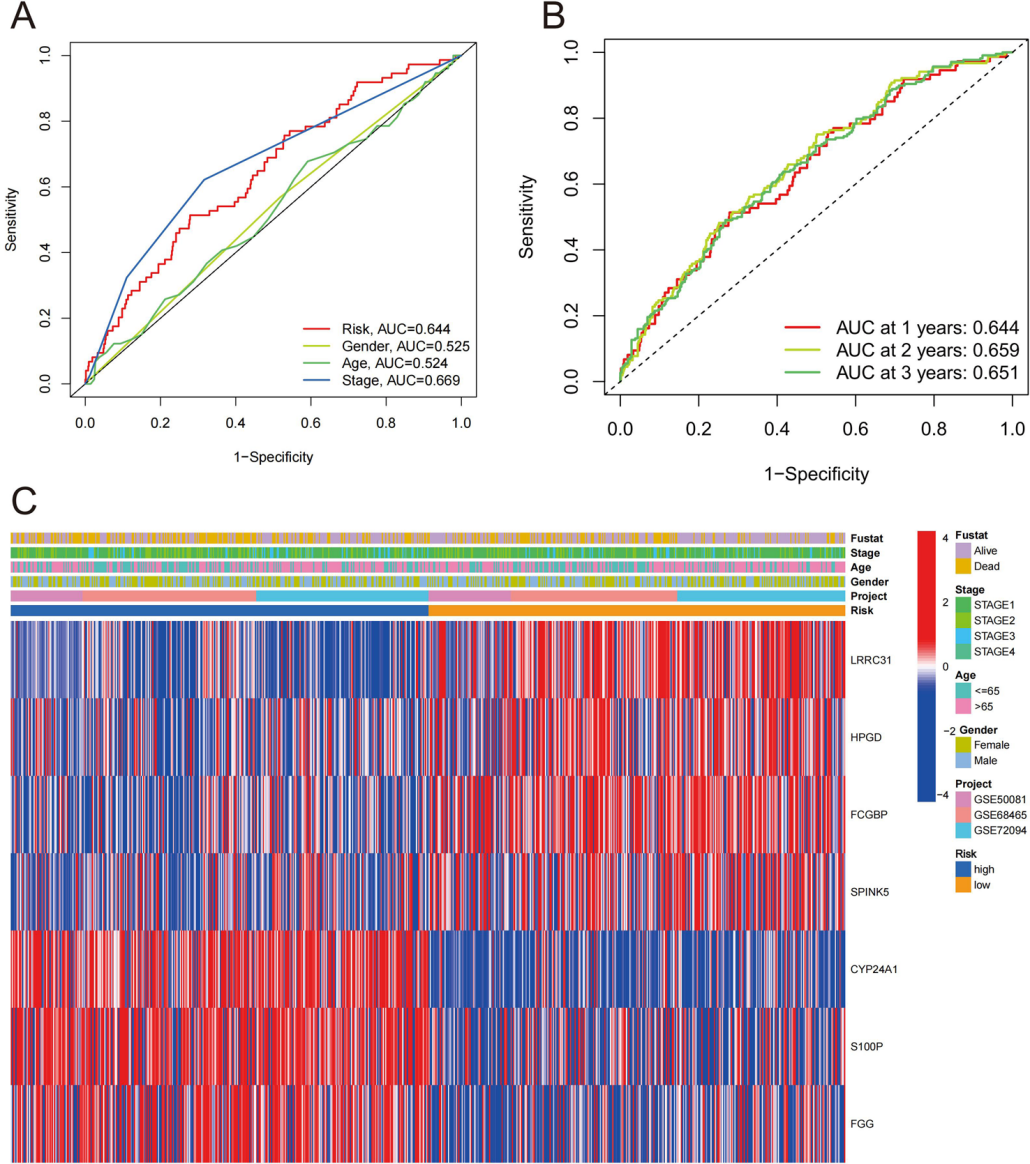
Clinical value analysis of the 7-gene risk signature. (**A**) ROC curves of clinical parameters and risk scores. (**B**) ROC curves of the 7-gene risk signature predicting the OS of 1-, 2-, and 3-year. (**C**) Heatmap illustrating correlations between clinical parameters and risk groups

ROC curve was first conducted to verify the reliability of the prognostic risk signature. Comparing different clinical parameters, the ROC value of the 7-gene risk signature was 0.644 (Fig. 4A). The area under curve (AUC) of predicting 1-, 2-, and 3-year survival rates were 0.644, 0.659, and 0.651, respectively (Fig. 4B). Meanwhile, we analyzed the clinical parameters of the risk group and constructed a heatmap based on the 7 prognostic smoking-related genes. (Fig. 4C). Applying Kaplan-Meier (K-M) survival analysis, we have stratified the clinical characteristics of HRG and LRG. Firstly, NSCLC patients with smoking history were divided into two stages by age: old stage (age > 65) and young stage (age ≤ 65). In two stages, HRG had worse OS compared with the LRG (Supplementary Fig. 1A, B). Next, according to gender, we found that HRG had a worse OS in both male and female groups (Supplementary Fig. 1C, D). Similarly, according to stage grade, samples of stage 1 and stage 2 were analyzed (Supplementary Fig. 1E, F). To sum up, stratified survival analysis indicated that the 7-gene risk signature had stable ability to predict prognosis in different clinical characteristics.

### Construction and evaluation of Nomogram

**Fig. 5:**
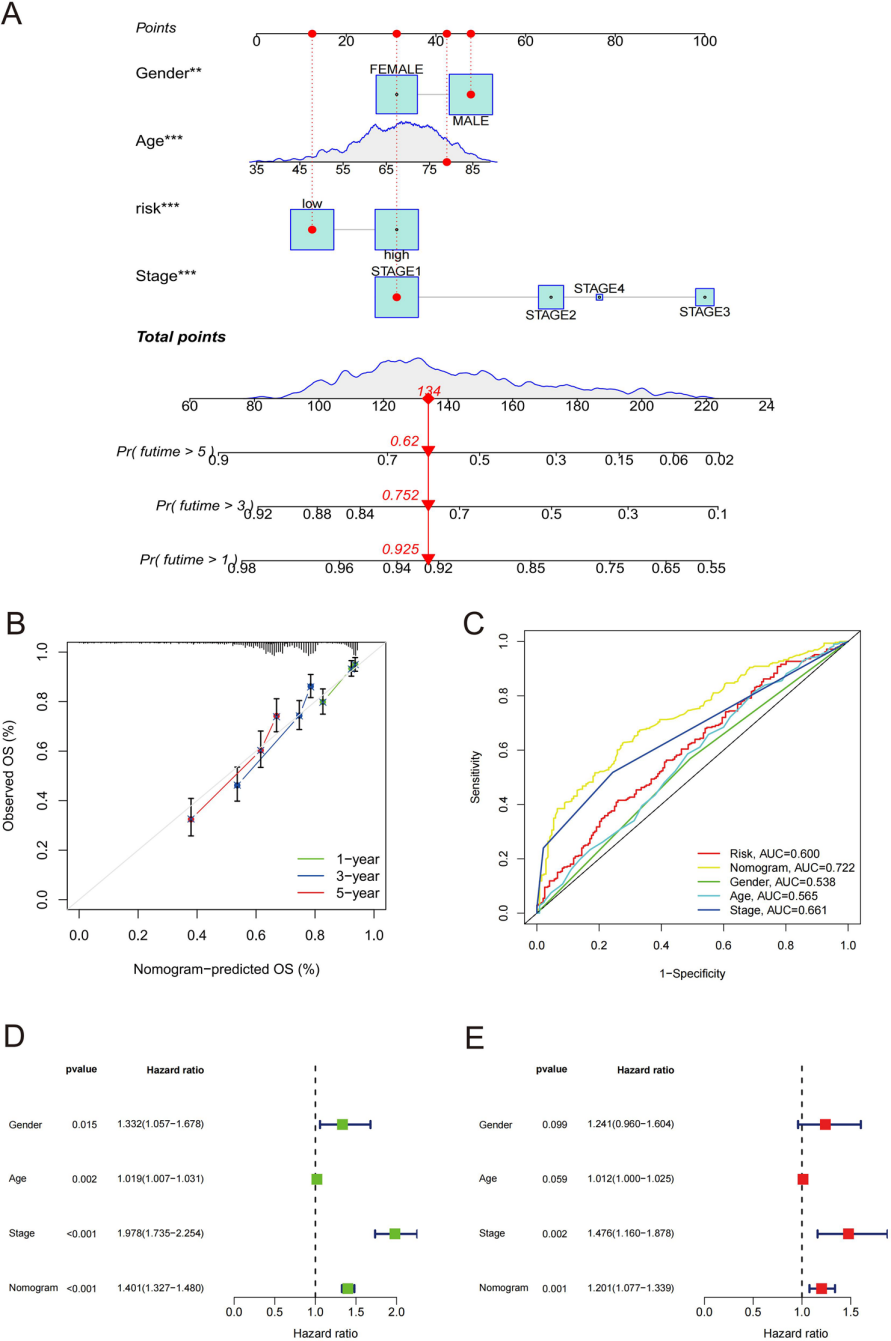
Construction and evaluation of nomogram. (**A**) Nomogram predicting 1-, 3-, and 5-year OS rate of NSCLC smokers. (**B**) Calibration curve for assessing the accuracy of the nomogram in predicting 1-, 3-, and 5-year OS rate. (**C**) ROC curves of clinical characteristics and the nomogram. (**D**, **E**) Univariate and multivariate Cox regression analysis of the nomogram. “*” *P* < 0.05, “**” *P* < 0.01, and “***” *P* < 0.001

In order to assess the risk of NSCLC patients with smoking history more accurately, we constructed a nomogram combining clinical feature and prognostic risk signature. According to the sum of relevant factors, the prediction probability of 1-, 3-, and 5-year survival rates were 0.925, 0.752, and 0.62, respectively (Fig. 5A). The calibration chart also showed the good prediction accuracy of the nomogram (Fig. 5B). Using ROC analysis, the AUC result of the nomogram was 0.722, which showed the best prediction accuracy compared with others clinical parameters (Fig. 5C). Cox univariate regression analysis indicated that the nomogram had relevance with NSCLC smokers’ OS (Fig. 5D). Moreover, according to multivariate Cox regression analysis, we found that the nomogram was an independent prognostic factor for forecasting the OS rates of NSCLC patients with smoking history in GSE50081, GSE68465 and GSE72094 cohort (Fig. 5E).

### Immune Microenvironment of 7-gene risk signature

**Fig. 6:**
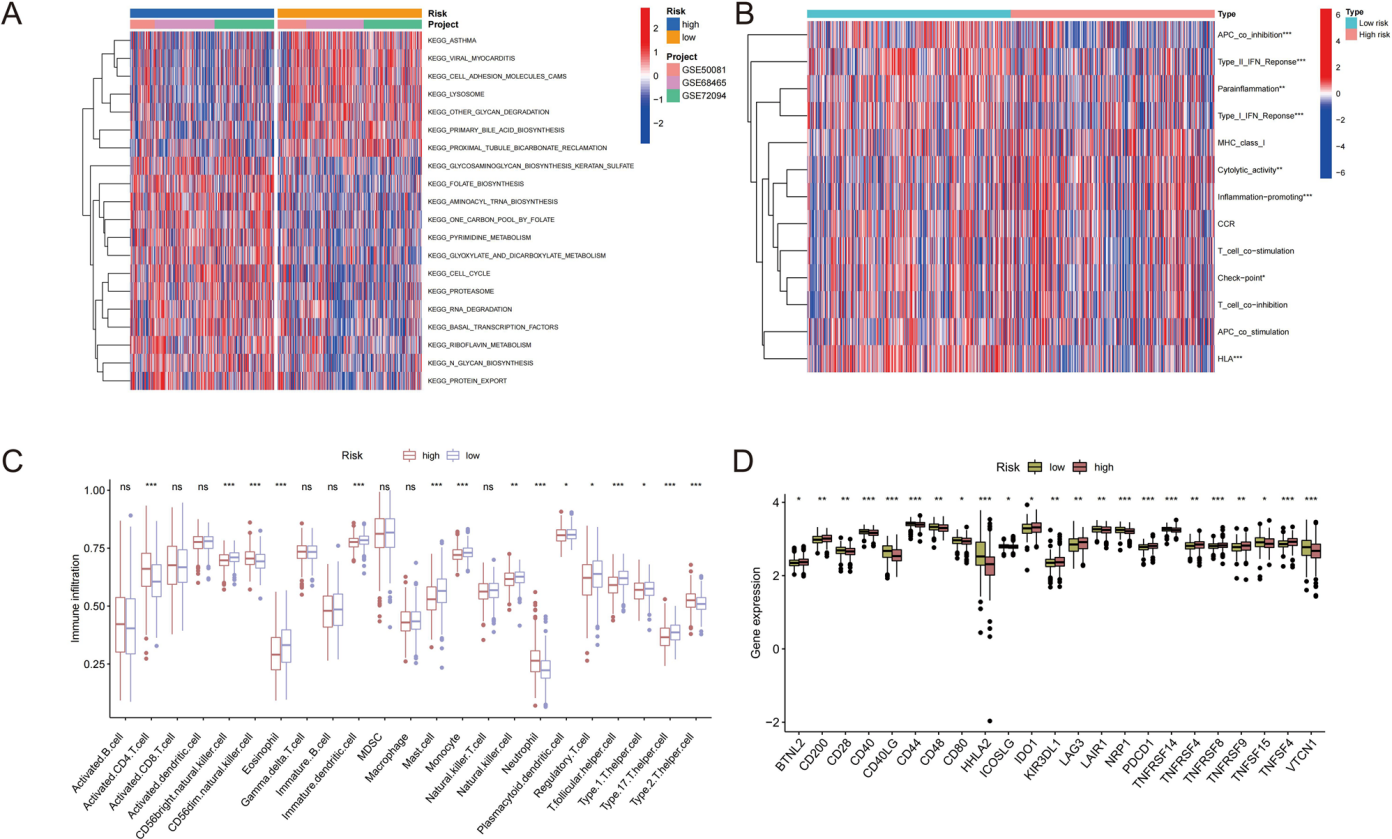
Immune-related analysis of HRG and LRG. (**A**) GSEA analysis showed significant pathway enrichment between HRG and LRG (**B**) The heatmap of immune function analysis results between HRG and LRG. (**C**) Boxplot of the infiltration degree of immune cells infiltration. (**D**) Differences in expression levels of 23 immune checkpoints between HRG and LRG. “*” *P* < 0.05, “**” *P* < 0.01, and “***” *P* < 0.001

Applying GSEA, we found that patients in LRG have better enrichment of immune activation and enrichment of patients in HRG more inclined to cellular mechanism such as cell cycle and RNA degradation (Fig. 6A). Subsequently, applying ‘limma’, ‘GSVA’, ‘GSEABase’, ‘pheatmap’ and ‘reshape2’ R package immune function analysis was performed to research difference of enrichment of immune pathways between HRG and LRG. At the same time, we visualized the enrichment results through heatmaps. In the heatmap, we found that tumor suppressor immune pathways such as APC co inhibition, Type II IFN response, Type I IFN response and HLA were significantly activated in LRG (Fig. 6B). In order to investigate the immune microenvironment, the infiltration degree of 23 immune cells in HRG and LRG was detected. Activated CD4 T cell, CD56dim natural killer cell, neutrophil and type 2 T helper cell, which interrelate with immunosuppression, had higher infiltration degree in HRG. In the other side, eleven kinds of immune cells interrelate with tumor immune activation were widely distributed in LRG, which indicate a better immune response of patients in LRG (Fig. 6C). Overall, LRG patients have better tumor immune activation, which explains why patients in LRG have better OS than those in HRG. In order to obtain better immunotherapy effect for patients in HRG, we explored the difference of immune checkpoints in two risk groups. 23 common immune checkpoint genes were found to be statistically different between the two risk groups. Patients in HRG had high expression of 11 kinds of immune checkpoint genes, including immunotherapy targets that have been proved effective in the treatment of lung cancer, such as *PDCD1* (*PD-1*), *TNFRSF8* (*CD30*), *IDO1* and *LAG3*. This discovery provides new possibilities for HRG patients to provide more effective immunotherapy strategies (Fig. 6D).

### Drug sensitivity analysis

To further explore the connection between the 7-gene risk signature and clinical medical treatment, we used the pRRophetic algorithm to assess drug susceptibilities by the half-maximal inhibitory concentration (IC50). By comparing the IC50 levels of HRG and LRG, 18 kinds of drugs were obtained. The analysis results show that patients in HRG have a higher sensitivity to common chemotherapy and immunotherapy drugs including sorafenib, doxorubicin, imatinib, midostaurin, pyrimethamine and vinorelbine, which make contributions to better prognosis of LRG patients (Supplementary Fig. 2A-M). Furthermore, rapamycin, MG-132, erlotinib, WZ-1-84 and Z-LLNle-CHO may be become new drugs to ameliorate the OS of LRG patients (Supplementary Fig. 2N-R).

### Validation of the 7-gene risk signature by the GSE29016 and GSE102287

**Fig. 7:**
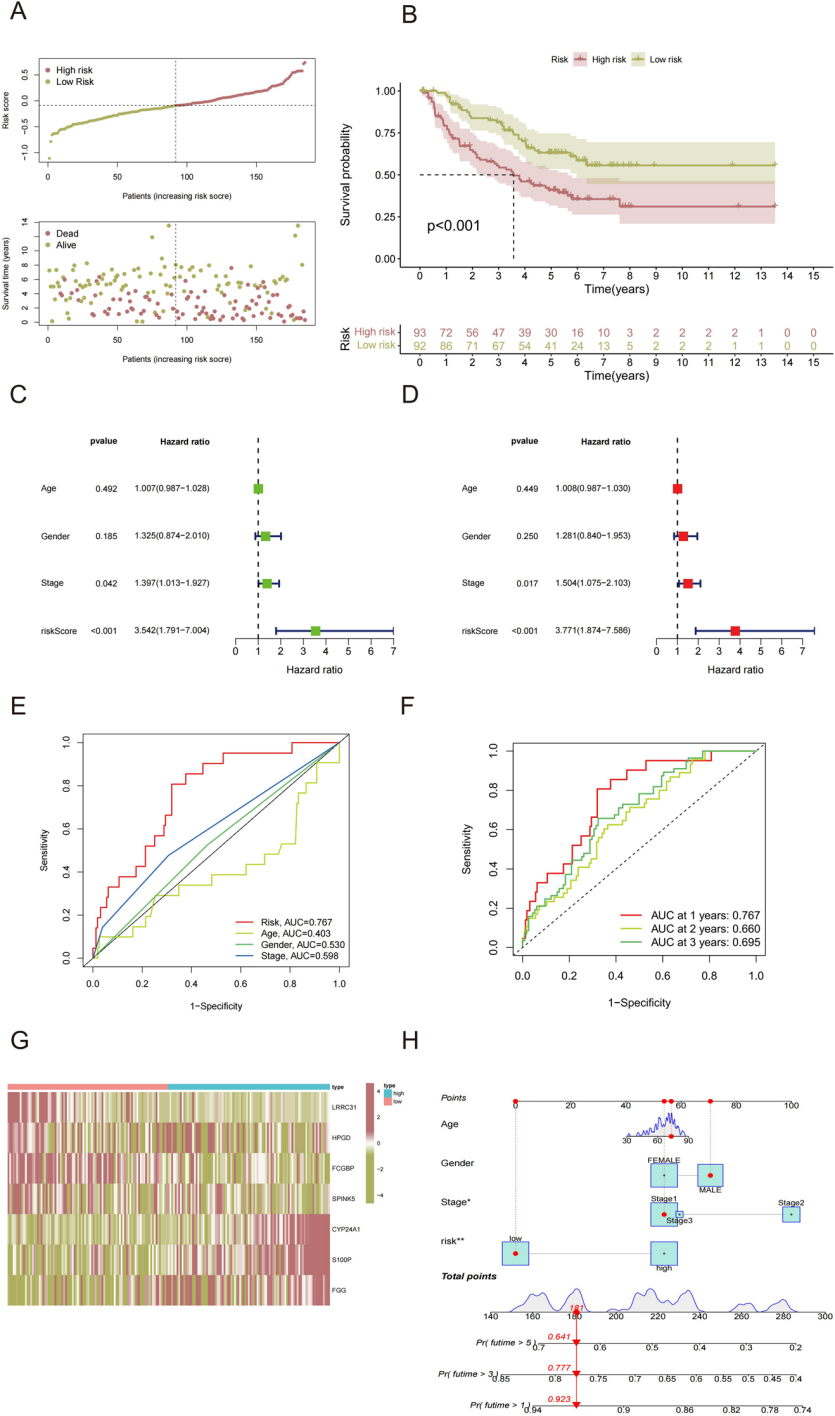
Validation of this 7-gene risk signature by the GSE29016 and GSE102287. (**A**) Risk score and survival status of NSCLC smokers in the GSE29016 and GSE102287. (**B**) Survival analysis of LRG and HRG. (**C**) Univariate Cox regression analysis and (**D**) multivariate Cox regression analysis of 7-gene risk signature. (**E**) ROC curves of clinical characteristics and 7-gene risk signature. (**F**) ROC curves indicate 7-gene risk signature forecasting the 1-, 2-, and 3-year OS. (**G**) Heatmap of the seven genes of LRG and HRG in external verification. (**H**) Nomogram based on 7-gene risk signature, age, gender, and TNM stage

To verify the stability of the 7-gene risk signature ‘s ability to predict the prognosis of NSCLC smokers, we established an external validation group by merging GSE29016 and GSE102287 and deleting unqualified patient data. The risk score of patients in external verification were gotten by the same formula. Same as mentioned above, HRG and LRG were distinguished according to the median of risk scores. Figure 7A visualized risk score and survival status’ distribution of patients in external verification. Survival analysis indicated that HRG patients had a worse OS compared the LRG group as expected (Fig. 7B). Univariate (Fig. 7C) and multivariate (Fig. 7D) cox regression analysis result showed that the 7-gene risk signature is an independent prognostic factor for predicting the OS of patients in external verification. ROC curve indicated that this risk signature predicted prognosis more accurately than other clinicopathological features in external verification (Fig. 7E). A time-dependent ROC curve showed the AUCs at 1, 2, and 3 years were 0.767, 0.660, and 0.695, respectively (Fig. 7F). The expression of seven prognostic smoking-related genes (*LRRC31*, *HPGD*, *FCGBP*, *SPINK5*, *CYP24A1*, *S100P* and *FGG*) in patients of external verification was shown by the heatmap (Fig. 7G). Ultimately, nomogram was constructed and the mortality rate of the patient in 1, 3 and 5 years was assessed to be 0.923, 0.777 and 0.641 (Fig. 7H). In summary, this 7-gene risk signature effectively predict the risk of NSCLC smokers in external verification, demonstrating the robust and stable predictive ability of this smoking-associated risk signature.

### Genetic Mutation and Survival Analysis of *FCGBP*

Owning to the close relationship between smoking and gene mutation, genetic mutation analysis was applied to explore mutation frequency of these seven prognostic smoking-related genes in NSCLC. With 12.85% mutation frequency in 1121 samples, 144 samples had prognostic smoking-related genes mutations. Among seven prognostic smoking-related genes, *FCGBP* carried the highest mutation frequency at 9%, so we further conducted further analysis on *FCGBP* (Supplementary Fig. 3A). Performing expression level analysis in patients, the expression level of *FCGBP* was found significantly downregulated in NSCLC patients with smoking history compared with non-smoking NSCLC patients (Supplementary Fig. 3B). Survival analysis indicated that the low expression of *FCGBP* interrelate with the deterioration of prognosis in smokers with NSCLC (Supplementary Fig. 3C).

### *FCGBP* Knockdown Promoting Proliferation, Migration, and Invasion in NSCLC Cells

**Fig. 8:**
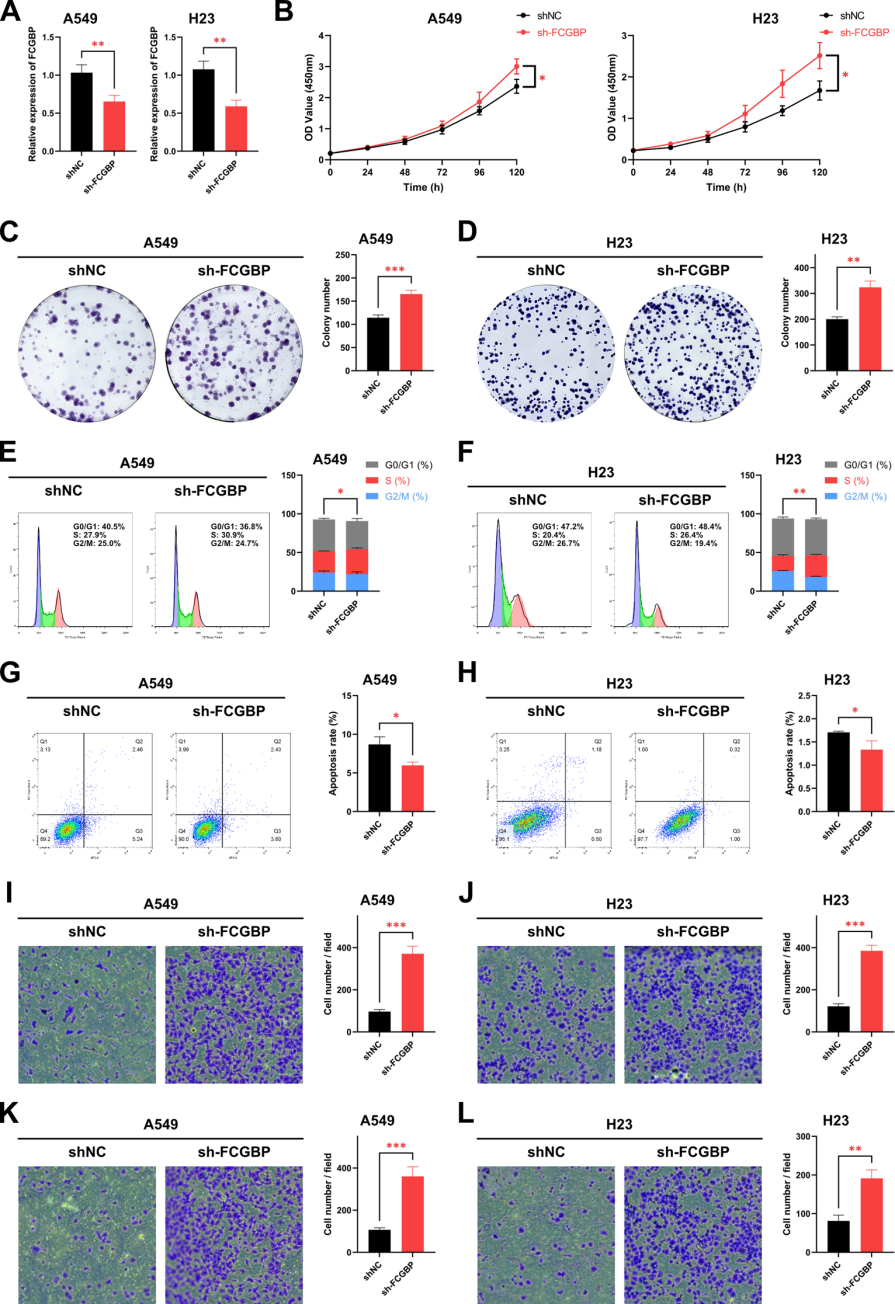
Exploring the effects of *FCGBP* knockout on LUAD cells through cell experiments (**A**) qPCR to investigate the knockdown efficiency of *FCGBP* in A549 and NCI-H23 cells. (**B**) CCK-8 assay to assess the effect of knockdown of *FCGBP* on the proliferation ability of A549 and NCI-H23 cells. (**C**, **D**) Compared with the siRNA negative control (NC) group, colony formation assay was applied to evaluate the effect of knockout of *FCGBP* of proliferative capacity in A549 and NCI-H23 cells. (**E**-**H**) Flow cytometry analysis was used to assess the effect of *FCGBP* knockdown on cell cycle and apoptosis. (**I**-**L**) Transwell analysis of migration and invasion ability of *FCGBP* knockdown in NSCLC cells

**Fig. 9:**
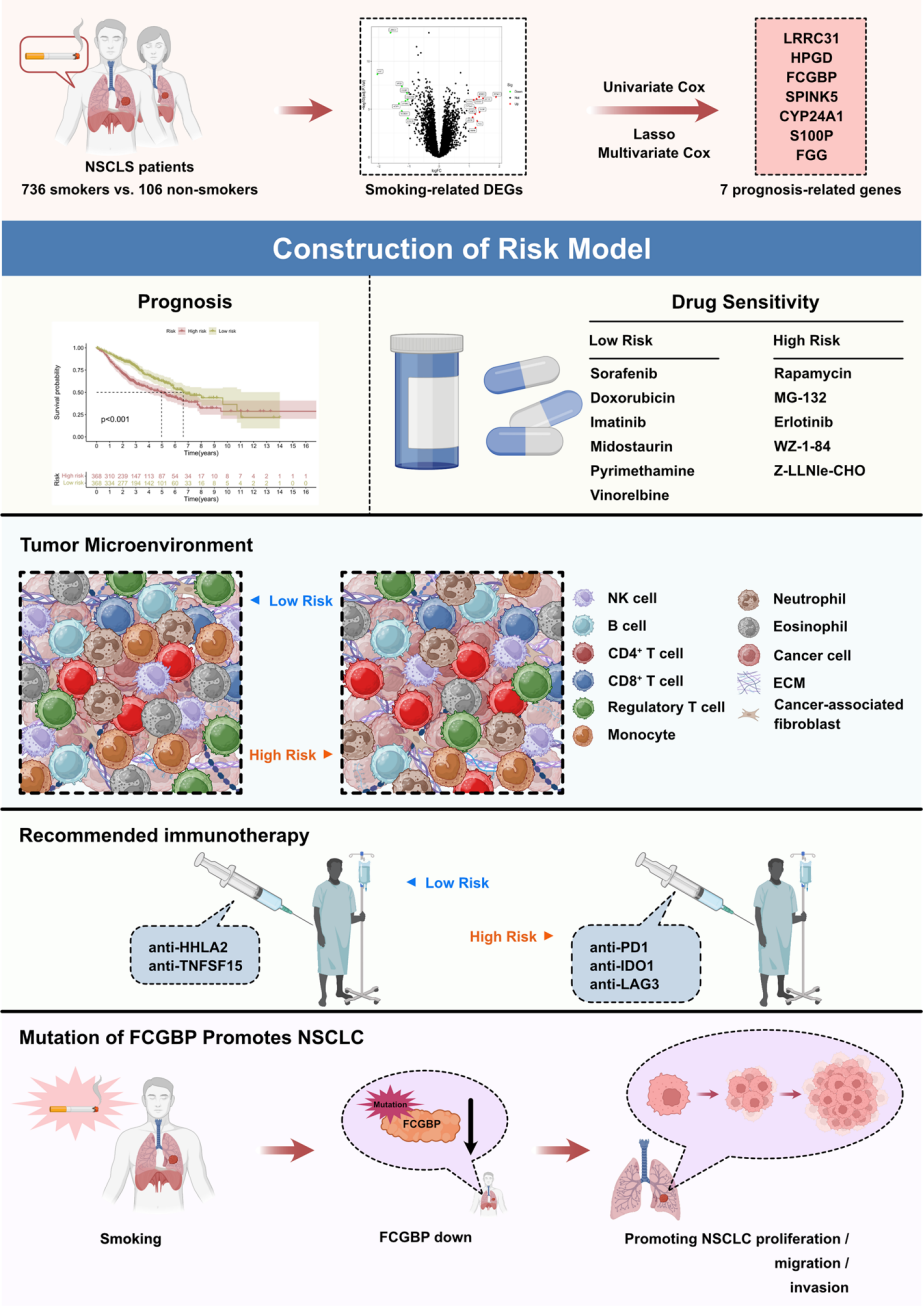
Summary diagram of this research work

Owning to smoking can cause *FCGBP* mutation and reduce the expression, we transfected *FCGBP* knockdown plasmids into A549 and NCI-H23 cells to investigate the effect of *FCGBP* knockdown in NSCLC cells (Fig. 8A). CCK-8 assays indicated that knockdown of *FCGBP* significantly promoted the growth rate of A549 and NCI-H23 cells (Fig. 8B). Compared with the control group, the colony forming units were significantly increased after *FCGBP* knockdown in A549 and NCI-H23 cells (Fig. 8C, D). Next, flow cytometry cell cycle analysis showed S-phase cells increased after *FCGBP* knockdown in A549 and NCI-H23 cells (Fig. 8E, F). In addition, flow cytometry demonstrated that apoptosis rate of *FCGBP* knockdown NSCLC cells significantly decreased (Fig. 8G, H). Applying transwell assays, we found that knockdown of *FCGBP* significantly strengthened the migration and invasion abilities of A549 and NCI-H23 cells (Fig. 8I-L). These experimental results support the above bioinformatics analysis conclusions and provide solid evidence for the rationality of selecting this prognostic model (Fig. 9).

## Discussion

As the most common subtype of lung cancer with high heterogeneity, NSCLC was known for its high mortality rate overall the world [20]. Due to clinical symptoms of NSCLC appear late, many patients are diagnosed at an advanced stage, thus missing the best treatment opportunity [21, 22]. Owning to gene changes precede obvious histopathological changes in cancer detection, there is an urgent need for a biomarker based on genetic aspect to predict the early survival prognosis, tumor microenvironment, immunotherapy targets and drug sensitivity of NSCLC [23, 24].

At present, smoking is regarded as the main cause of lung cancer, and nearly 90% of lung cancer can be attributed to smoking [25]. Smoking will induce multiple DNA damage, inhibit DNA repair and cause gene changes, thus promoting the occurrence and development of lung cancer [26]. Many studies have shown that NSCLC of non-smokers and NSCLC of smokers have different molecular biology [27, 28]. The development of gene sequencing technology makes it possible to identify some potential genes with predictive value in NSCLC patients with smoking history and non-smoking NSCLC patients [29, 30]. However, there are few reliable biomarkers to help diagnosis. Therefore, it is necessary to identify smoking-related genes and construct robust risk signature to help early diagnosis of NSCLC patients.

In this research, we identified 20 differentially expressed smoking-related genes by analyzing the mRNA expression profiles of genes between NSCLC patients with smoking history and NSCLC samples without smoking history. In order to accurately quantify and assess smoking-related genes in NSCLC, Lasso Cox regression was applied to build a prognostic risk model. By calculating the risk score, samples were classified into HRG and LRG. Patients in HRG showed worse outcomes (*P* < 0.001). ROC curve and nomogram are regarded as reliable tools to forecasting the prognosis of cancer [31–33]. ROC curves and nomogram demonstrated the 7-gene signature had high accuracy in farecasting the prognosis of NSCLC patients with smoking history. Furthermore, stratified analysis showed that the 7-gene prognostic signature maintained a stable predictive ability in terms of age, gender and stage. In addition, higher immune cell infiltration that promote tumor immunity was found in LRG, such as *CD56* bright natural killer cell, eosinophil, mast cell, and T follicular helper cell [34–37]. The analysis of immune pathway also indicated that patients in LRG have better antitumor immune activation, which contribute to better outcomes. A significant connection between the risk signature and immunotherapy was found, which indicated this signature provides new possibilities for HRG patients to provide more effective immunotherapy strategies. Last but not least, we conducted IC50 analysis to evaluate drug sensitivity and screen drugs to provide reference for future treatment.

The risk signature is composed of seven prognostic smoking-related genes (*LRRC31*, *HPGD*, *FCGBP*, *SPINK5*, *CYP24A1*, *S100P* and *FGG*). Among these genes, *LRRC31*, *HPGD*, *FCGBP* and *SPINK5* are served as protected factors. In the other hand, *CYP24A1*, *S100P* and *FGG* are found as risk factors. Previous research confirmed the accuracy of our research. *LRRC31* was found to be a DNA repair suppressor that can target cancer radiation to increase sensitivity [38]. Overexpression of *HPGD* inhibited the proliferation, migration and anchoring growth of cervical cancer cells [39]. *SPINK5* plays a tumor inhibitor role in NSCLC by negatively regulating *PSIP1* [40]. Low expression of *CYP24A1* is correlated with poor prognosis in breast cancer [41]. *S100P* was found to increase the migration and invasion of cancer cells in lung cancer [42]. *FGG* regulates the expression of *SLUG* and *ZEB1*, and promotes the migration and invasion of hepatocellular carcinoma cells through *EMT* signal pathway [43]. IgG Fc-binding protein (*FCGBP*) was found to be closely related to mutations caused by smoking [44]. Applying genetic mutation analysis, *FCGBP* had the highest mutation frequency among seven prognostic smoking-related genes, which is identified as the key gene of smoking mutation. *FCGBP* has been proven to participate in intestinal tumor immunity [45]. However, the role of *FCGBP* in NSCLC has not been researched. We found that *FCGBP* was significantly downregulated in NSCLC patients with smoking history compared with non-smoking NSCLC patients. Cell experiments demonstrated that *FCGBP* knockdown promoting proliferation, migration, and invasion in A549 and NCI-H23 cell lines. This shows that the low expression of *FCGBP* caused by smoking can promote the progress of NSCLC, which provides a novel screening biomarker and treatment targets for NSCLC smokers.

This study also had some noteworthy limitations. On the one hand, more NSCLC samples are needed to maintain the reliability of smoking-related prognostic signature. On the other hand, further research on upstream and downstream pathway of *FCGBP* in NSCLC is necessary.

## Conclusion

To sum up, we built a prognostic risk model based on seven prognostic smoking-related genes, which can accurately evaluate the prognosis, immunotherapy, drug sensitivity and tumor microenvironment of NSCLC patients. Importantly, the role of *FCGBP* in NSCLC was detected by cell experiments, which provides a screening biomarker and therapeutic target for NSCLC.

### Electronic supplementary material

Below is the link to the electronic supplementary material.

Supplementary Material 1: **Fig. S1** Stratified clinicopathological characteristics analysis

Supplementary Material 2: **Fig. S2** Drug sensitive prediction

Supplementary Material 3: **Fig. S3** Mutation and survival analysis of *FCGBP* in NSCLC. (**A**) Mutation frequency of seven prognostic smoking-related genes in 1121 NSCLC samples. (**B**) Expression level of *FCGBP* in NSCLC smoker and non-smoking NSCLC patients. (**C**) The prognosis of NSCLC smokers with low expression of *FCGBP* is significantly worse than that of smokers with high expression of *FCGBP*

## Data Availability

Not applicable.
