# Supplementary figures and images for: A novel prognostic signature based on smoking-associated genes for predicting prognosis and immune microenvironment in NSCLC smokers
Source: Cancer Cell Int. 2024 May 15;24:171. doi: 10.1186/s12935-024-03347-9 (PMC11094918; doi:10.1186/s12935-024-03347-9)

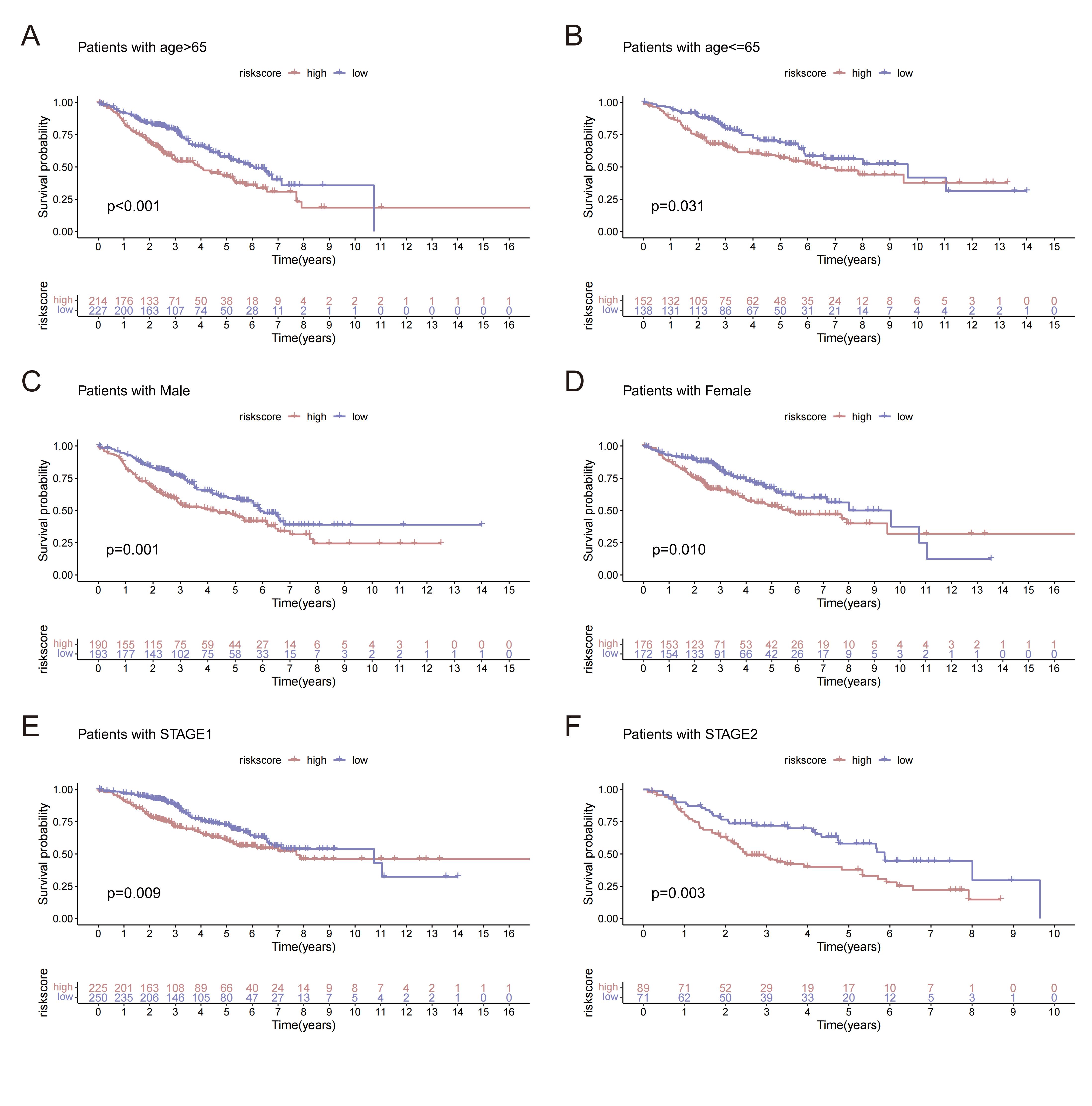

Supplement: Supplementary file 1 — Supplementary Material 1: Fig. S1 Stratified clinicopathological characteristics analysis [file 12935_2024_3347_MOESM1_ESM.jpg]

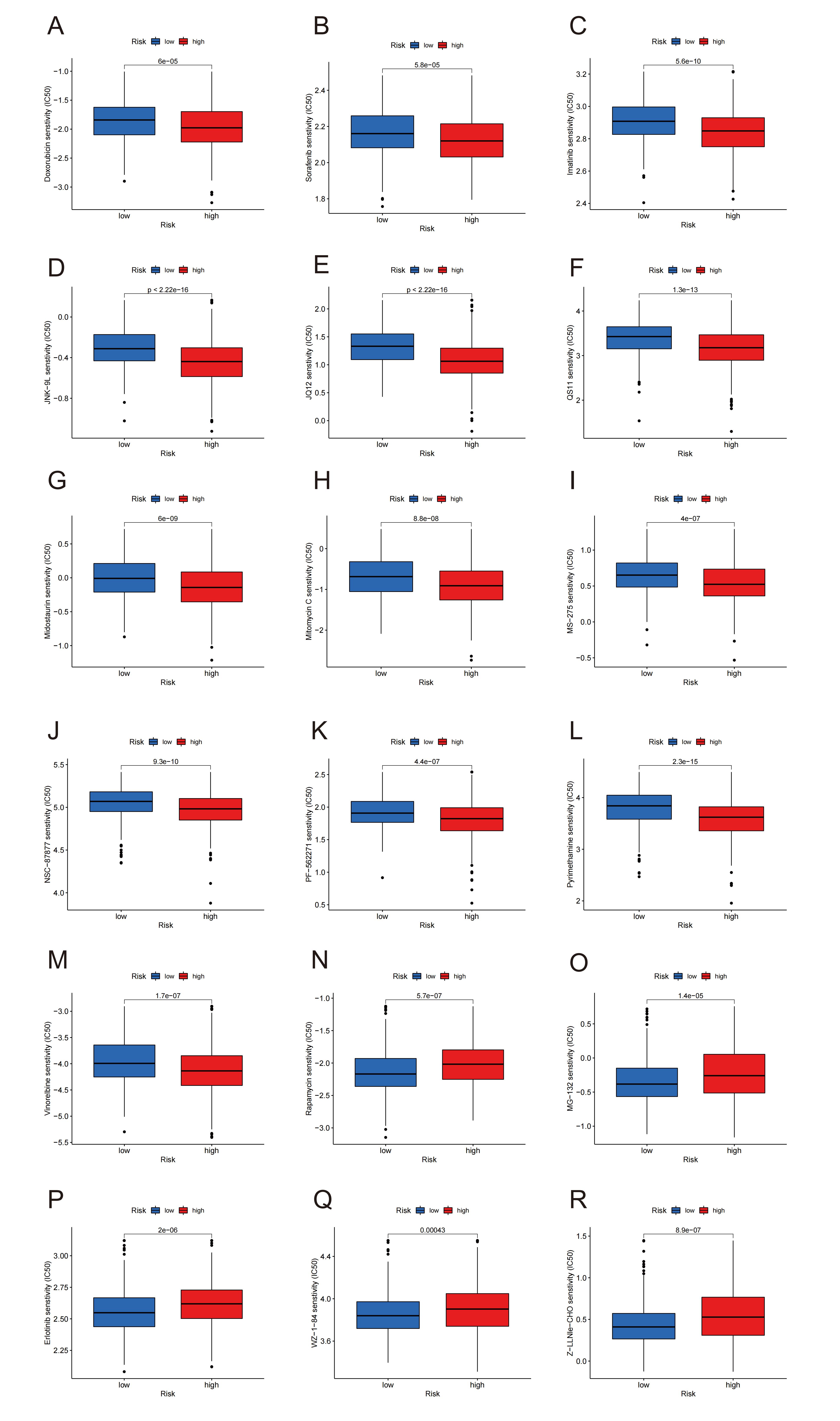

Supplement: Supplementary file 2 — Supplementary Material 2: Fig. S2 Drug sensitive prediction [file 12935_2024_3347_MOESM2_ESM.jpg]

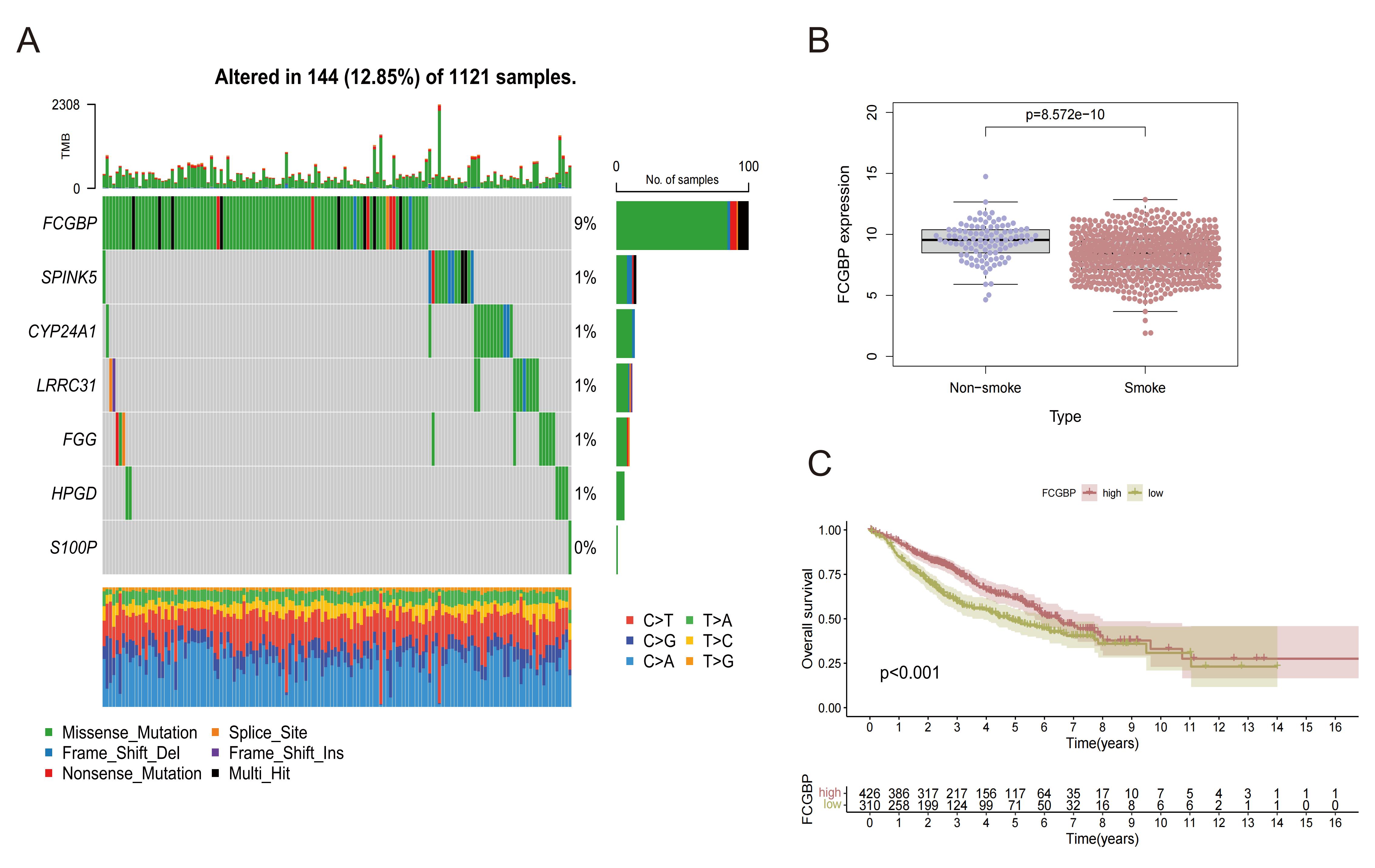

Supplement: Supplementary file 3 — Supplementary Material 3: Fig. S3 Mutation and survival analysis of FCGBP in NSCLC. (A) Mutation frequency of seven prognostic smoking-related genes in 1121 NSCLC samples. (B) Expression level of FCGBP in NSCLC smoker and non-smoking NSCLC patients. (C) The prognosis of NSCLC smokers with low expression of FCGBP is significantly worse than that of smokers with high expression of FCGBP [file 12935_2024_3347_MOESM3_ESM.jpg]
